# Emergency Roux-en-Y Biliary Bypass and Duodenal Exclusion for Perforated Duodenal Ulcer

**DOI:** 10.7759/cureus.100948

**Published:** 2026-01-06

**Authors:** Alexandra Z Zalums, Henry Hook

**Affiliations:** 1 Department of General Surgery, Orange Health Service, Orange, AUS

**Keywords:** comorbid, duodenal exclusion, gastroenterostomy, perforated duodenal ulcer, roux-en-y bypass

## Abstract

Perforated duodenal ulcers are a life-threatening surgical emergency with high morbidity and mortality; however, there is a paucity of literature describing the operative management of perforated ulcers involving the common bile duct (CBD). This manuscript describes the case of a woman in her 60s with multiple comorbidities who underwent emergency abdominal surgery for a large perforated duodenal ulcer with extension into the CBD. An intraoperative decision was made to perform a Roux-en-Y biliary bypass with duodenal exclusion and gastroenterostomy. Her immediate postoperative course was complicated by a transient bile leak and hospital-acquired pneumonia. She was discharged with lifelong proton pump inhibitor therapy and smoking cessation counseling. Although one-month follow-up imaging confirmed an intact reconstruction, she developed malnutrition and a large upper gastrointestinal bleed secondary to a marginal ulcer two months postoperatively. This technique offers a viable salvage option when conventional repair is not possible but is associated with high morbidity.

## Introduction

Surgical management of perforated duodenal ulcers, particularly those that are large or located near the common bile duct (CBD), represents a major surgical challenge associated with high morbidity and mortality. In 2019, the global prevalence of peptic ulcer disease (PUD) was approximately 8.09 million. In Australia, the age-standardized prevalence rate across both sexes was 15-40 per 100,000 population [1]. Perforated peptic ulcer (PPU) is the most frequent indication for emergency surgery for PUD and has an average 30-day mortality rate of 23.5% [2]. It is associated with high postoperative morbidity, with 50-60% of patients experiencing complications [3].

Patients typically present to hospital or primary care settings with severe, acute-onset epigastric abdominal pain, dehydration, and shock. Urgent intravenous fluid resuscitation, antibiotics, and surgical review are essential in the initial management [2]. The complexity of managing perforated duodenal ulcers stems from the difficulty of repairing large perforations (often greater than 2 cm, sometimes called “giant perforations”) that involve major tissue loss, especially when located in the D1 and/or D2 segments [4]. The ideal surgical treatment for acute duodenal injuries should be definitive while maintaining low morbidity and mortality [4].

A recent systematic review of etiologies and outcomes of duodenal perforation in acute peritonitis found that treatment approaches varied depending on the cause and timeliness of treatment, with significant postoperative complications noted [5]. Traditional simple surgical measures, such as primary repair or omental patch, often yield poor outcomes and high rates of persistent duodenal fistula in cases of large perforations [4]. One definitive strategy for managing major duodenal perforations described in the literature is pancreas-sparing, ampulla-preserving duodenectomy. The goal of this technique is to preserve the pancreas, ampulla of Vater, and the CBD to avoid the high morbidity and mortality associated with performing an emergency Whipple procedure (pancreatoduodenectomy) in a patient with acute peritonitis [2,4]. Other principles of surgical management of these complex cases include pyloric exclusion, external biliary diversion, and Roux-en-Y reconstruction [5]. There is a paucity of literature describing extrahepatic biliary diversion and the creation of a Roux-en-Y limb for large, complex PPUs; however, it is a recognized method for draining the extrahepatic biliary tree, particularly after major ductal injury.

## Case presentation

Clinical presentation

A woman in her 60s from rural New South Wales, Australia, was transferred to our emergency department from a small rural hospital with severe, acute-onset abdominal pain and vomiting. She had initially been admitted for an infective exacerbation of chronic obstructive pulmonary disease (COPD), for which she received intravenous antibiotics and steroid therapy, when she suddenly deteriorated, developing severe epigastric abdominal pain and vomiting. She was urgently transferred to our larger regional hospital for assessment and surgical review, as these resources were not available at the smaller rural hospital.

This occurred in the context of prior triple-negative breast cancer treated with mastectomy and chemotherapy two years earlier, cholecystectomy, COPD, hypertension, a single left kidney, epilepsy, and chronic lower back pain. She was an active smoker with a 50-pack-year smoking history. On arrival, she was hemodynamically stable, saturating at 96% on 2 L low-flow nasal-prong oxygen. She had upper abdominal tenderness with guarding. Her presentation was concerning for an intra-abdominal pathology, prompting urgent surgical review and further investigation.

Investigations

**Figure 1:**
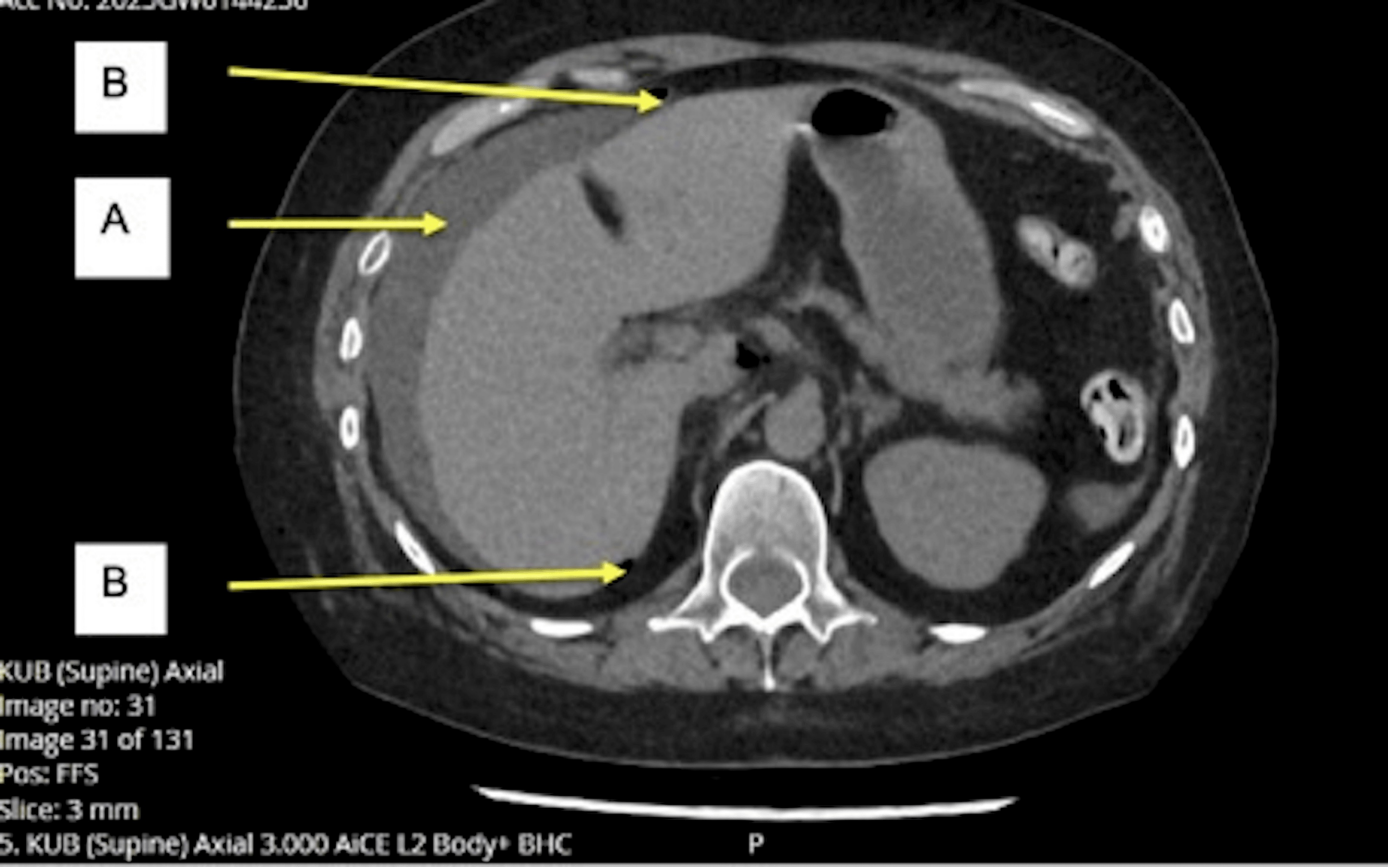
Axial slice of CT KUB demonstrating a moderate volume of free intraperitoneal fluid (A) and locules of extraluminal air (B) in the upper abdomen KUB, kidneys, ureters, and bladder

**Figure 2:**
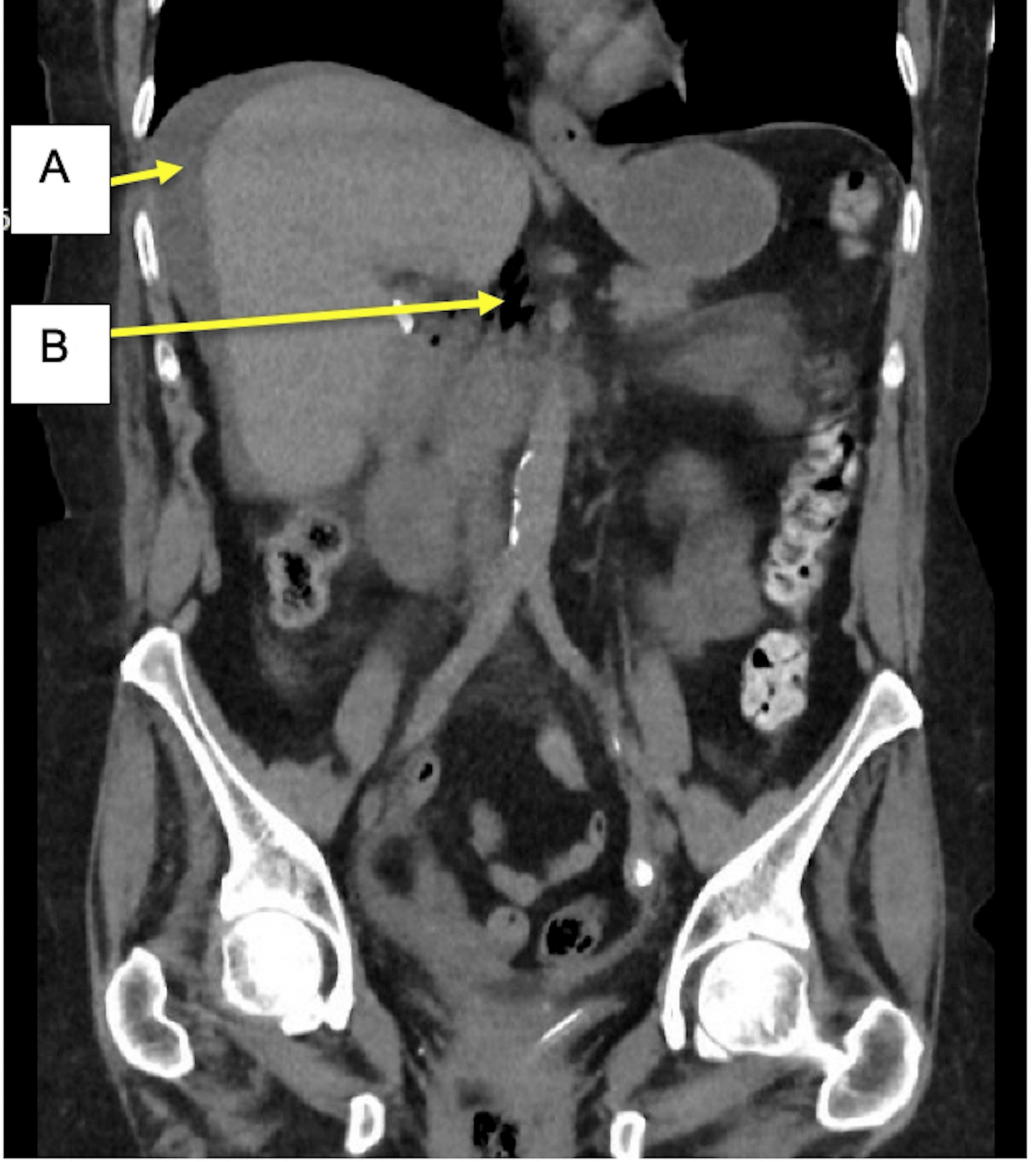
Coronal slice of CT KUB demonstrating a moderate volume of perihepatic intra-abdominal free fluid (A) and locules of extraluminal air (B) in proximity to the duodenum KUB, kidneys, ureters, and bladder

She underwent blood tests demonstrating an acute kidney injury (AKI), with an estimated glomerular filtration rate of 18 mL/min/1.73 m² and a creatinine level of 237 mmol/L. She had raised inflammatory markers, with a C-reactive protein level of 84 mg/L and a white cell count of 14 × 10⁹/L. A decision was made to perform a CT scan of the kidneys, ureters, and bladder to investigate the cause of the AKI and avoid the administration of intravenous contrast. This demonstrated scattered free gas in the upper abdomen and a moderate volume of free fluid in the upper abdomen, with stranding around the third part of the duodenum, consistent with a perforated hollow viscus (Figure 1, Figure 2). Given the presence of free air, the usual limitation of noncontrast CT, namely, its high false-negative rate for detecting peptic ulcer disease, did not prevent reaching an urgent diagnosis.

Diagnosis

Integrating the clinical, biochemical, and radiological features described above, a provisional diagnosis of a perforated viscus, likely a peptic ulcer, was made. The AKI was consistent with a prerenal insult from vomiting and gastric contents leaking freely into the abdomen and was more likely given her single kidney. She had significant risk factors for PUD, including a heavy smoking history and recent corticosteroid use.

Treatment

**Figure 3:**
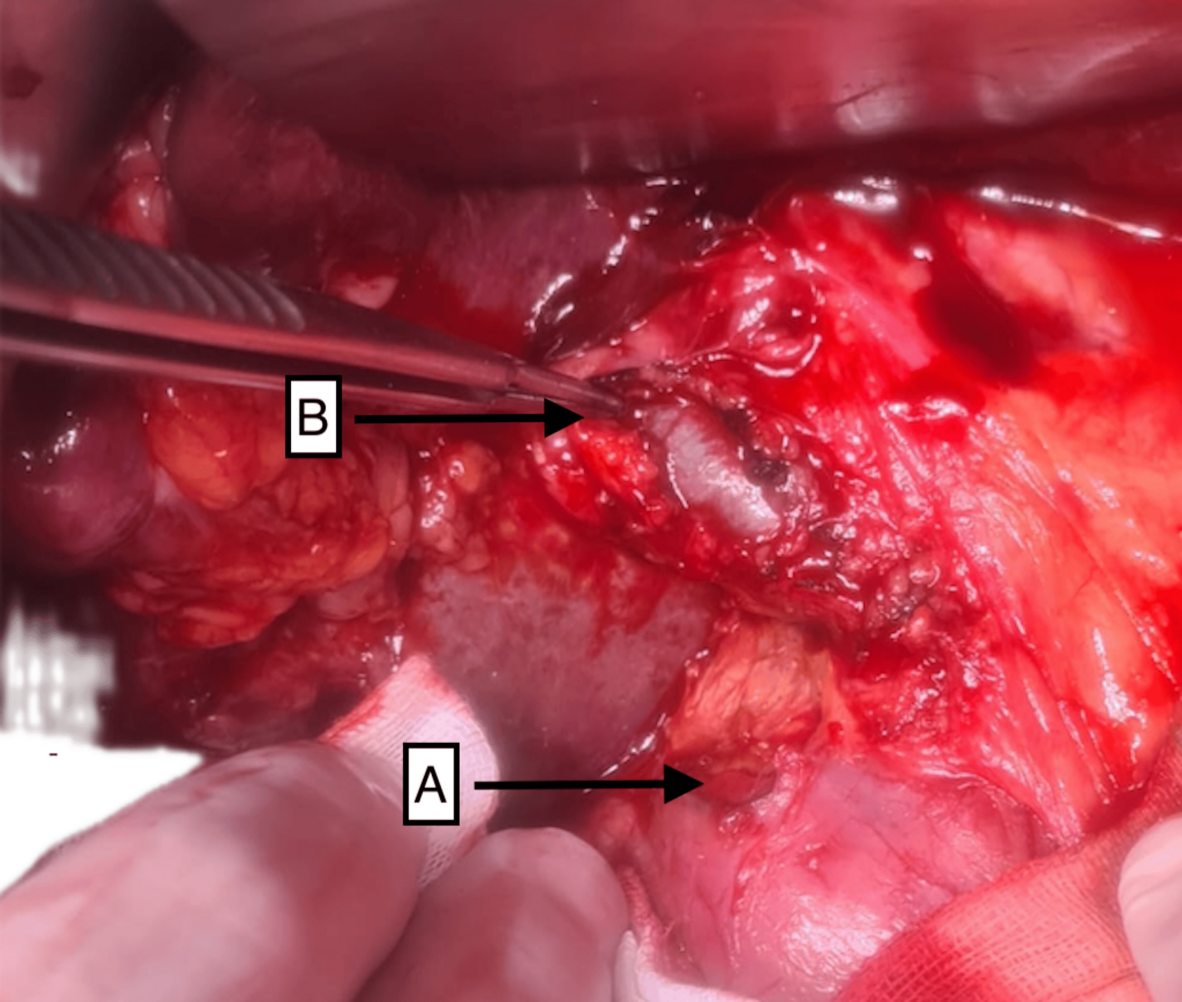
Intraoperative photograph of the upper abdomen revealing a giant ulcer in the first part of the duodenum (A) and involvement of the lateral CBD, which is being pointed to with forceps and has been dissected and slung (B) CBD, common bile duct

Following diagnosis, she was urgently transferred to the operating theater for emergency laparoscopy converted to laparotomy with oversewing of the duodenal ulcer, Roux-en-Y biliary bypass, duodenal exclusion, and gastroenterostomy. During laparoscopy, extensive bile-stained free fluid, fibrin, and pus were found in the upper abdomen. There were adhesions to the gallbladder fossa and midline requiring adhesiolysis. A large perforated D1 ulcer with extension into the lateral wall of the lower CBD was identified (Figure 3). There was no duodenal margin adjacent to the CBD; therefore, primary repair was not feasible without injury to the lateral wall.

**Figure 4:**
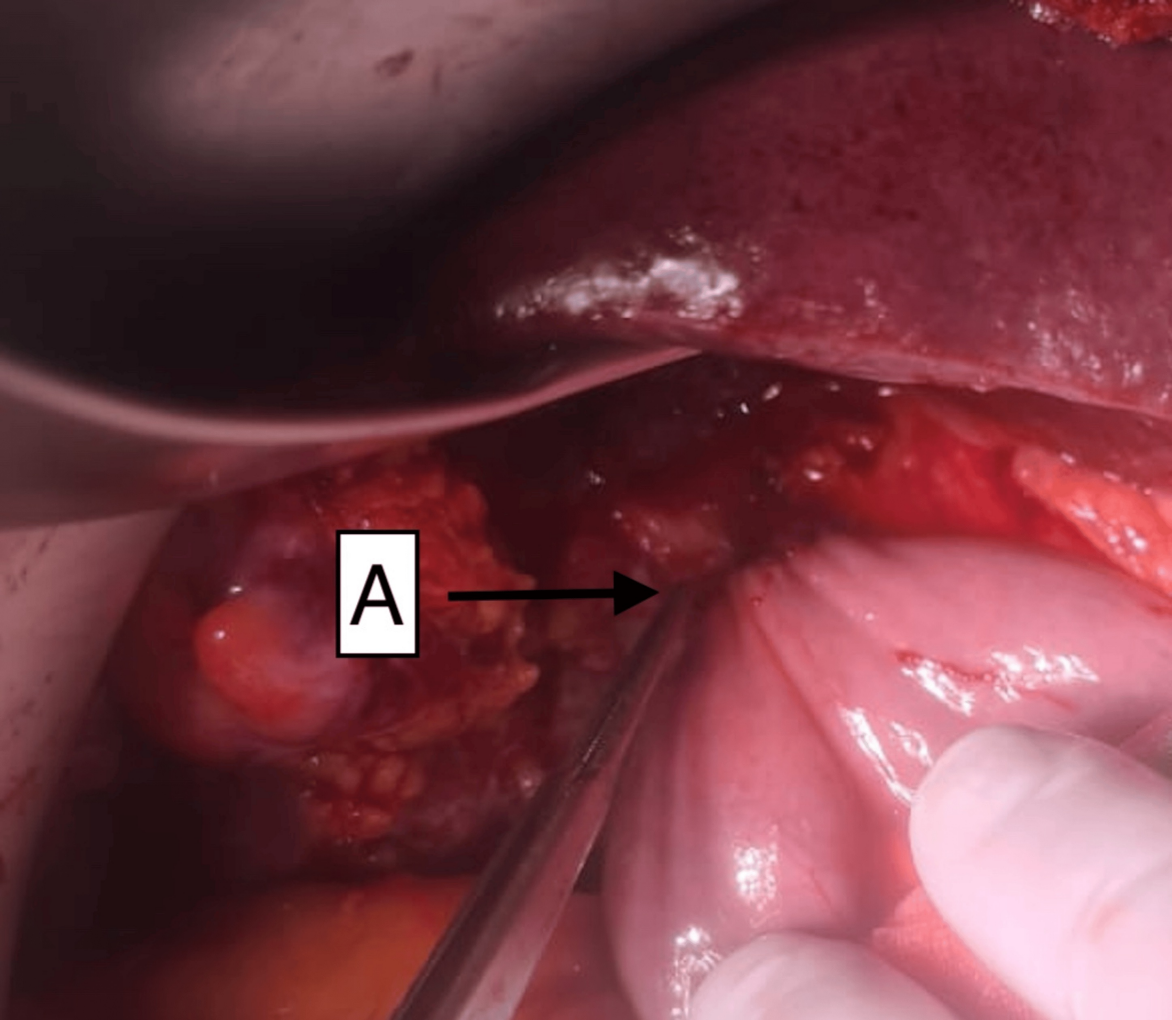
Intraoperative photograph of the upper abdomen demonstrating formation of a hepaticojejunostomy joining the proximal common hepatic duct to the Roux limb of the jejunum (A)

A decision was made to convert to a midline laparotomy, with a Thompson retractor used to expose the first part of the duodenum and hepatoduodenal ligament. The proximal CBD was dissected and slung, then divided just below the hilum, with ligation of the distal end using 0 Vicryl. The D1 defect was oversewn with interrupted 3-0 polydioxanone (PDS II) sutures. A noncutting stapler was fired across the distal antrum of the stomach to exclude the duodenum. The jejunum was divided approximately 20 cm from the duodenojejunal flexure. The Roux limb was brought through a retrocolic route to the right of the middle colic vessels. A handsewn hepaticojejunostomy was performed using interrupted 4-0 Monocryl parachute end-to-side sutures (Figure 4). Additional interrupted 4-0 PDS sutures were placed anteriorly and posteriorly at areas of bile staining at the anastomosis on check swabs. 

**Figure 5:**
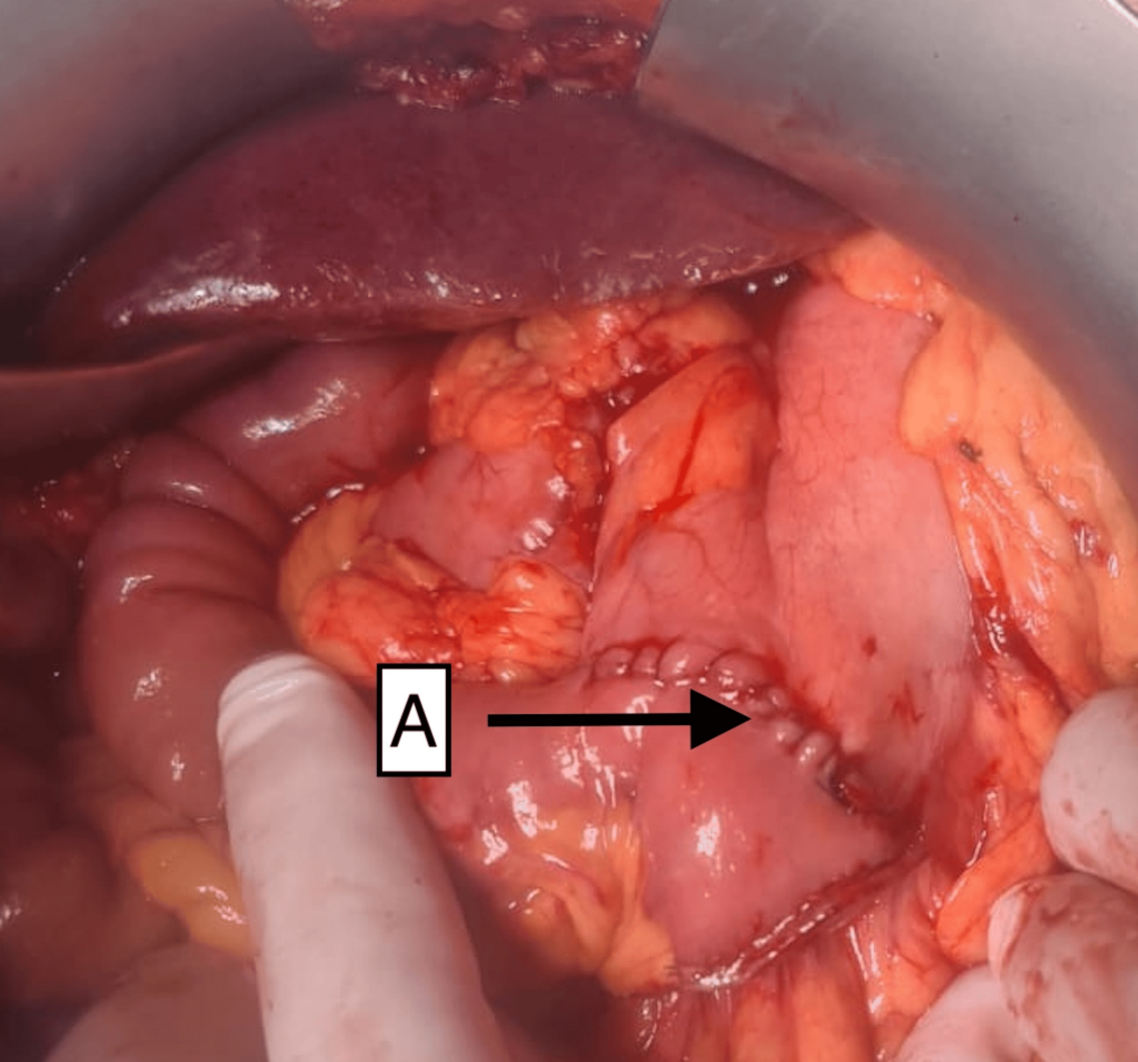
Intraoperative photograph demonstrating final reconstruction of the anatomy with biliary diversion and continuity of the alimentary tract through a handsewn gastrojejunostomy (A)

**Figure 6:**
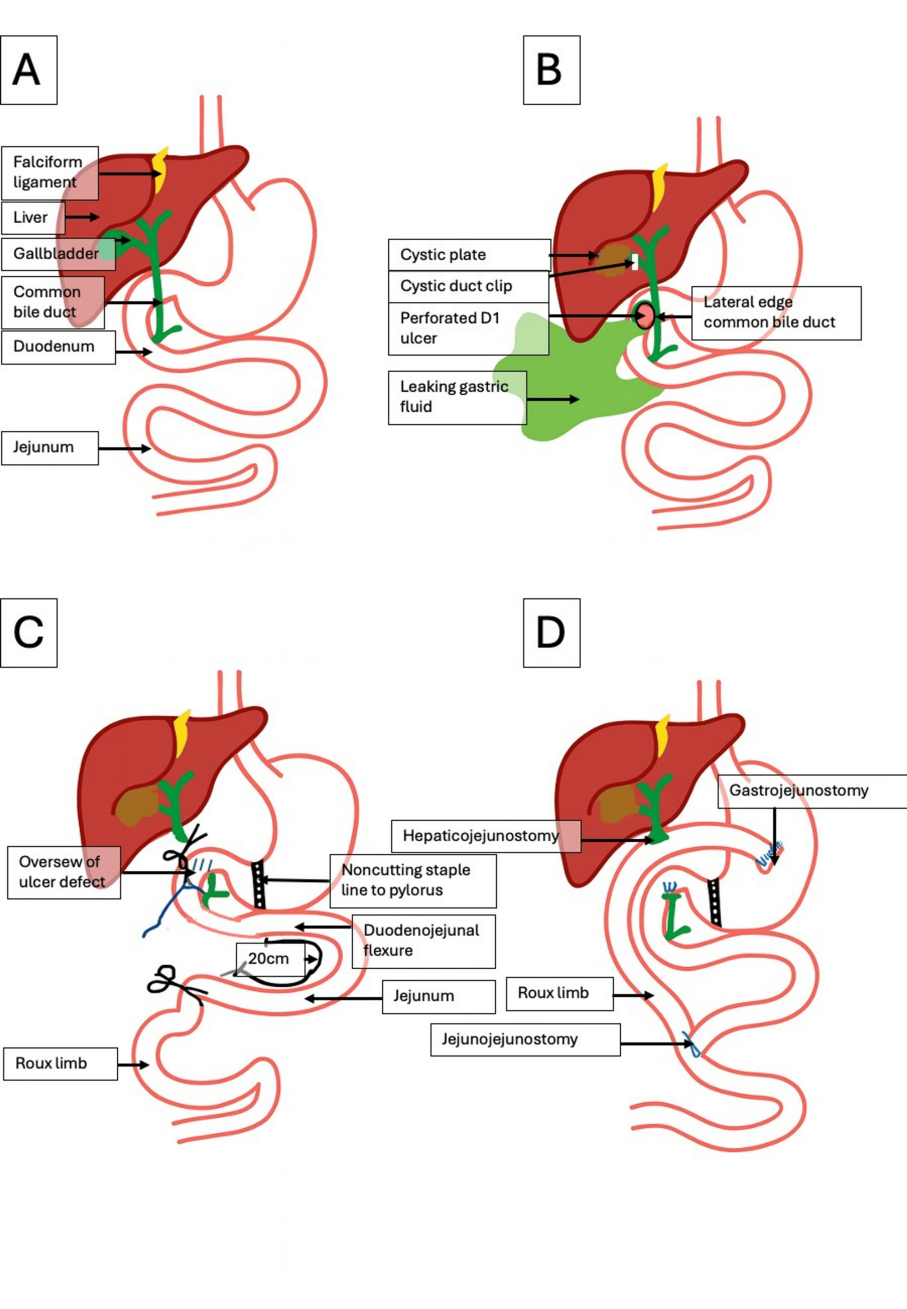
Illustration depicting normal anatomy (A), preoperative anatomy specific to the case (B), intraoperative components of the operation (C), and final reconstructed anatomy (D) (A) Normal biliary and alimentary anatomy. (B) Patient’s prior cholecystectomy state with a cystic duct clip in situ, a giant perforated ulcer in the first part of the duodenum (D1) involving the lateral aspect of the CBD, and subsequent leakage of gastric contents. (C) Components of the operation, including oversewing of the ulcer, suture ligation of the distal CBD, placement of a noncutting staple to exclude the pylorus, and division of the jejunum approximately 20 cm from the duodenojejunal flexure to create the Roux limb. (D) Final reconstruction and restoration of the alimentary tract with a hepaticojejunostomy, gastrojejunostomy, and jejunojejunostomy. CBD, common bile duct

A handsewn gastrojejunostomy was performed using a continuous single-layer 3-0 PDS side-to-side technique (Figure 5). A handsewn jejunojejunostomy was created with a single-layer 3-0 PDS suture; however, the distal 1 cm of the bypass limb appeared dusky, so it was resected, and the jejunojejunostomy was redone. The intraoperative findings, operative steps, and final reconstruction are summarized in the illustration below (Figure 6). Extensive lavage of the abdomen with normal saline was performed, and a 19-Fr Blake drain was placed in the subhepatic space. The abdomen was closed with 1 PDS loop suture, skin clips, and Monocryl to port sites. 

She was admitted to the intensive care unit postoperatively, intubated, and on metaraminol. She was extubated on postoperative day 2, requiring high-flow nasal-prong oxygen, and the noradrenaline was weaned. She had high nasogastric aspirates, requiring commencement of total parenteral nutrition (TPN). She developed hypoactive delirium. On postoperative day 3, she developed a bile leak, and intravenous proton pump inhibitor (PPI) therapy and antibiotics were commenced. By postoperative day 5, the bile leak had resolved, her bowels were functioning, and nasogastric aspirates were minimal. She was weaned to low-flow nasal-prong oxygen and stepped down to the general surgical ward.

On postoperative day 6, she developed a new fever and tachypnea, with a chest radiograph demonstrating new hospital-acquired pneumonia (HAP) and atelectasis. She was commenced on intravenous piperacillin-tazobactam, a respiratory consult was obtained, and chest physiotherapy was initiated. She also developed a right upper arm occlusive cephalic vein thrombus, requiring commencement of rivaroxaban anticoagulation, as recommended by the hematology team. She subsequently progressed well in hospital, with gradual advancement of diet, weaning of TPN, completion of treatment for HAP, and continued progress with physiotherapy and dietitian input. She was discharged to a peripheral site for ongoing physiotherapy and dietitian support for management of deconditioning and weight loss and was discharged home on postoperative day 19.

Outcome and follow-up

She presented to the emergency department three times in the two months following discharge with intermittent upper abdominal pain, anorexia, and weight loss. At the first presentation, she was evaluated for cardiac causes, which were negative. A CT scan of the abdomen and pelvis demonstrated pneumobilia with an intact reconstruction. She was discharged with dietary supplementation, oral analgesia, and surgical follow-up.

At the second presentation, she underwent CT mesenteric angiography, which demonstrated critical stenosis of the celiac artery with collateral distal supply. Vascular surgery input was sought, and she was commenced on aspirin and cholesterol-lowering medication. At routine surgical follow-up, she was found to be minimally compliant with recommended acid-suppression medical therapy and was failing to thrive at home, with minimal oral intake, chronic upper abdominal discomfort, and further weight loss. It was recommended that she be admitted to the hospital for upper gastrointestinal endoscopy, insertion of a nasojejunal feeding tube, and commencement of enteral nutrition with electrolyte monitoring.

While awaiting endoscopy in the hospital, she developed a massive upper gastrointestinal bleed (UGIB), requiring resuscitation with a massive transfusion protocol and emergency endoscopy. She was found to have a large nonbleeding marginal ulcer, which was injected with adrenaline and treated with hemospray. She was commenced on high-dose PPI therapy and sucralfate. Nasojejunal feeds were initiated, and she was supported through a period of refeeding syndrome with intravenous electrolyte replacement and close dietitian involvement. The follow-up period ended at this time following a patient-led discharge, despite medical recommendations to continue nasojejunal feeds.

## Discussion

PPU remains the most frequent indication for emergency surgery in PUD, despite the declining incidence associated with widespread PPI use and *Helicobacter pylori *eradication therapy [3]. In 2019, the global prevalence of PUD was estimated at 8.09 million, with an Australian age-standardized prevalence rate of 15-40 per 100,000 [1]. Although most perforations can be managed with simple closure, giant or complex duodenal perforations, particularly those involving adjacent structures such as the CBD, pose unique technical challenges, with no clear consensus on optimal management [2].

In the present case, a woman in her 60s with significant comorbidities and recent steroid and antibiotic exposure developed a perforated duodenal ulcer extending into the lateral wall of the CBD. This case highlights the multifactorial nature of PUD, in which smoking and steroid use are recognized risk factors [2]. The proximity of the perforation to the CBD precluded simple closure or Graham patch repair due to the risk of ductal injury. This presentation represents an exceptionally uncommon scenario, with few published reports describing emergency reconstruction involving both pyloric exclusion and Roux-en-Y biliary-enteric diversion in the setting of a PPU.

Operative strategy and justification

According to the World Society of Emergency Surgery guidelines, management of giant duodenal perforations (>2 cm) should be tailored to the anatomical site and local tissue condition. Definitive resection is generally discouraged in the setting of peritonitis and hemodynamic instability due to high morbidity and mortality [2]. Instead, damage-control approaches, such as pyloric exclusion, external biliary diversion, and gastric decompression, are recommended as safer alternatives.

In this case, intraoperative findings revealed extensive bile peritonitis and a large D1 perforation extending into the lateral CBD wall, with friable tissue and adhesions in the hepatoduodenal region. The absence of a viable duodenal margin adjacent to the CBD rendered primary repair impossible without risking ductal injury. Conversion from laparoscopy to open laparotomy allowed for safe exposure and complex reconstruction. The chosen strategy of duodenal exclusion with Roux-en-Y hepaticojejunostomy and gastrojejunostomy achieved source control and restored biliary and alimentary continuity while avoiding direct manipulation of the injured duodenal wall [6].

Comparison to existing literature

Hudnall et al. emphasized that while most PPUs can be closed primarily, large or destructive defects may require resection and reconstruction, with Roux-en-Y gastrojejunostomy being a favorable method due to reduced bile reflux and improved nutritional outcomes [6]. Similarly, Di Saverio et al. described duodenectomy and Roux-en-Y reconstruction for major D1/D2 duodenal injuries near the ampulla of Vater, underscoring the importance of complete resection or diversion when the duodenal wall and periampullary region are compromised [4].

However, the present case differs fundamentally in that the perforation extended into the CBD, necessitating Roux-en-Y biliary-enteric reconstruction rather than duodenectomy alone. According to Chassin’s description of Roux-en-Y biliary-enteric bypass, this procedure is indicated in settings of CBD obstruction or iatrogenic injury to re-establish biliary continuity [7]. In their systematic review, Shahi et al. highlight the importance of tailoring the surgical technique during exploratory laparotomy for duodenal perforation to the individual patient’s circulatory status and local tissue condition [5]. To our knowledge, this approach has not previously been described as part of the emergency management of a PPU involving the biliary tree. The operative plan mirrored established principles of safe bile duct reconstruction, such as proximal duct division, end-to-side hepaticojejunostomy, and creation of a tension-free Roux limb, adapted to a contaminated, inflamed field.

Vu et al. reported an emergency conversion to Roux-en-Y gastric bypass for staple-line leaks, noting the significant technical complexity and risk of anastomotic complications associated with performing Roux-en-Y reconstructions in emergency rather than elective settings [8]. This observation aligns with the technical and postoperative challenges encountered in the present case, including the early bile leak, respiratory complications, and eventual UGIB secondary to marginal ulcer formation. The patient’s immediate postoperative course was ultimately favorable, with successful resolution of the bile leak by postoperative day five and recovery without further intra-abdominal complications. However, longer-term issues with anorexia, malnutrition, and marginal ulcer formation remain challenging and contribute to a high degree of morbidity.

Contributing factors and broader implications

Multiple predisposing factors may have contributed to the development and severity of the ulcer perforation in this patient, including active smoking, chronic steroid use during the recent COPD exacerbation, and possibly impaired mucosal defense due to prior chemotherapy and chronic illness [3]. The presence of a single kidney and preexisting renal impairment further complicated perioperative management, necessitating careful balance of contrast exposure, fluid resuscitation, and postoperative nutrition through TPN.

This case underscores several important considerations. Preoperative recognition of atypical perforation is critical. CT findings of retroperitoneal gas or biliary tract involvement should raise suspicion for complex duodenal pathology. Operative flexibility is essential in adapting standard ulcer repair techniques to complex anatomical scenarios. Damage-control principles, including pyloric exclusion, Roux-en-Y diversion, and staged nutritional support, can be lifesaving and allow for safe recovery even in frail or comorbid patients.

## Conclusions

Effective surgical management of large PPUs is significantly influenced by predisposing patient factors, complex duodenal anatomy, and staged postoperative nutritional support. Salvage options are required in emergency settings where standard closure, patch repair, or resection are not feasible, such as in ulcers involving the CBD, where surgical flexibility is imperative. Despite the complexity and high risk of morbidity, definitive biliary-enteric reconstruction can be safely achieved in selected emergency cases when guided by sound anatomical principles and damage-control philosophy. Morbidity following biliary-enteric reconstruction in the emergency setting is influenced by preexisting patient factors, postoperative nutritional support, and adherence to preventive strategies such as smoking cessation, acid-suppression therapy, and optimization of existing health conditions.
